# Evaluation of the Deletion of African Swine Fever Virus E111R Gene from the Georgia Isolate in Virus Replication and Virulence in Domestic Pigs

**DOI:** 10.3390/v16091502

**Published:** 2024-09-23

**Authors:** Elizabeth Ramirez-Medina, Lauro Velazquez-Salinas, Alyssa Valladares, Amanda Meyers, Leeanna Burton, Ediane Silva, Jason Clark, Manuel V. Borca, Douglas P. Gladue

**Affiliations:** 1 U.S. Department of Agriculture, Agricultural Research Service, Foreign Animal Disease Research Unit, Plum Island Animal Disease Center (PIADC), P.O. Box 848, Greenport, NY 11944, USA; lauro.velazquez@usda.gov (L.V.-S.); alyssa.valladares@usda.gov (A.V.); amanda.meyers@usda.gov (A.M.); leeanna.burton@usda.gov (L.B.); ediane.silva@usda.gov (E.S.); jason.clark@usda.gov (J.C.); 2 U.S. Department of Agriculture, Agricultural Research Service, Foreign Animal Disease Research Unit, National Bio and Agro-Defense Facility, Manhattan, KS 66502, USA; 3 Oak Ridge Institute for Science and Education (ORISE), Oak Ridge, TN 37830, USA

**Keywords:** ASFV, ASF, African swine fever virus, recombinant virus, ASFV virulence, ASFV E111R gene

## Abstract

African swine fever virus (ASFV) is the causative agent of an often lethal disease in domestic pigs, African swine fever (ASF). ASF is currently a pandemic disease challenging pig production in Eurasia. While the ASFV genome encodes for over 160 proteins, the function of most of them are still not characterized. Among those ASF genes with unknown functions is the E111R gene. It has been recently reported that the deletion of the E111R gene from the genome of the virulent Chinese field isolate SY18 strain produced a reduction of virus virulence when pigs were inoculated at relatively low doses. Conversely, we report here that deletion of the ASFV gene E111R in the Georgia 2010 isolate does not alter the virulence of the parental virus in experimentally inoculated pigs. A recombinant virus lacking the E111R gene, ASFV-G-∆E111R was intramuscularly (IM) inoculated in domestic pigs at a dose of 10^2^ HAD_50_ of ASFV-G-∆E111R and compared with animals that received a similar dose of virulent ASFV-G. Both, animals inoculated with either the recombinant ASFV-G-∆E111R or the parental virus developed a fatal form of the disease and were euthanized around the 6th–7th day post-inoculation (dpi).

## 1. Introduction

African swine fever virus (ASFV) infects domestic and wild swine, causing a disease, African swine fever (ASF), that can present in a variety of clinical forms depending on the acting virus strain and the characteristics of the infected host [1]. ASF was mostly restricted to the sub-Saharan African region but since 2007, has widely spread across Eurasia and has just been discovered in the Dominican Republic and Haiti after more than 40 years of being absent in the Western hemisphere [1]. The disease has severely affected pig production and is causing food shortages worldwide.

ASFV is a highly structurally complex virus, harboring a large double-stranded DNA genome of approximately 180–190 kilobase pairs encoding more than 150 genes [2]. Most of these genes have not been characterized, particularly in their role in basic virus functions such as replication and virulence in the natural hosts. The development of recombinant viruses harboring deletions of specific genes is widely accepted as an effective tool to study gene function within the homologous virus field. Therefore, in the last years, several ASFV genes have been studied following this approach [3]. For instance, the identification of genes involved in virus virulence in pigs has been a critical initial step in designing of live attenuated ASFV vaccine candidates [4,5,6,7]. Among those poorly characterized ASFV genes is E111R, whose function remains unknown. It has been recently reported that E111R could be involved in the process of virus virulence [8]. Recombinant viruses harboring a deletion of E111R in the genome of the virulent Chinese isolate SY18 have shown a significant reduction of virulence when inoculated in domestic pigs at a relatively low dose, 10^2^ HAD_50_. Conversely, here we report that a recombinant virus lacking the E111R gene in the highly virulent Georgia isolate (ASFV-G) is as virulent as its parental virus. The possible causes of the differences in the effect of deletion of the E111R gene between these two virulent and genetical similar field isolates is further discussed.

## 2. Materials and Methods

### 2.1. Viruses and Cells

Primary cultures of swine macrophages, prepared as previously described [9], were used at a density of 5 × 10^6^ cells per mL. ASFV Georgia (ASFV-G) is a field isolate kindly provided by Dr. Nino Vepkhvadze from the Laboratory of the Ministry of Agriculture (LMA) in Tbilisi, Republic of Georgia [10]. Comparative growth curves between ASFV-G-∆E111R and the parental ASFV-G were conducted in primary swine macrophage cell cultures using an MOI of 0.01 HAD_50_ (hemadsorbing doses, as previously determined in primary swine macrophage cell cultures) [11]. Sample points were taken at 2, 24, 48, 72, 96, and 120 h post-infection; cells were frozen at ≤−70 °C and then thawed; and the cell lysates were titrated using primary swine macrophage in 96-well plates. The presence of virus-infected cells was assessed by hemadsorption (HA), and virus titers were calculated as previously described [12].

### 2.2. Development of the ASFV E111R Gene Deletion Mutant

A recombinant ASFV harboring a deletion of the E111R gene (ASFV-G-∆E111R) was developed by homologous recombination between the genome of the parental ASFV-G and a recombination transfer vector as previously described [13]. The recombinant transfer vector (named p72mCherryΔE111R) comprises the genomic regions flanking the E111R gene: the left arm extends for approximately 1000 base pairs toward the left of nucleotide position 170066 and the right arm approximately 1000 base pairs to the right from nucleotide position 170401, harboring a reporter gene cassette that includes the mCherry fluorescent protein (mCherry) gene under the control of the ASFV p72 late gene promoter [14]. The recombinant transfer vector was obtained by DNA synthesis (Epoch Life Sciences, Sugar Land, TX, USA). As designed, this construction created a complete deletion of the E111R gene (between nucleotide positions 170067–170402). Recombinant ASFV-G-∆E111R was purified by consecutive limiting dilution steps based on mCherry activity detection. The stock of ASFV-G-∆E111R was full-length-sequenced using next-generation sequencing (NGS).

### 2.3. Next Generation Sequencing of ASFV

Virus DNA was extracted from infected cell cultures, reaching 90–100% CPE, using a nuclear extraction kit (Active Motif cat# 40010). The nucleus and cytoplasmic fractions were separated, and the cytoplasmic fraction was used to isolate the viral DNA, following the manufacturer’s protocol. Briefly, infected cells were treated with hypotonic buffer for 15 min. on ice. The nucleus fraction was then separated by centrifugation. DNA was extracted from the remining cytoplasmic fraction by adding 10% 3M NaOAc by volume to the sample (Sigma-Aldrich, St. Louis, MO, USA) and an equal volume of phenol:chloroform:isoamyl alcohol (25:24:1) with a pH of 6.5–6.9 (Sigma-Aldrich P3803-100 ML) and centrifuged at maximum speed in a tabletop centrifuge. The aqueous layer was then ethanol-precipitated using 2 volumes of 100% ethanol, washed with the same volume of 70% ethanol, and dried. The resulting DNA pellet was then reconstituted in sterile water. Then, this DNA library was used for NGS sequencing utilizing the Nextera XT kit in the NextSeq (Illumina, San Diego, CA, USA) following the manufacturer’s protocol. Sequence analysis was performed using CLC Genomics Workbench software 23.0.4 (CLCBio, Waltham, MA, USA).

### 2.4. Detection of E111R Transcription

As previously described [5], we used a real-time PCR assay (qPCR) to evaluate the transcriptional profile of the E111R gene during the infection of ASFV-G in cultures of porcine macrophages, using the early CP204L (p30) and late B646L (p72) expressed genes of ASFV as reference genes. Briefly, cell cultures of porcine macrophages were infected with a stock of ASFV-G using an MOI of 1. RNA extractions using an RNeasy Kit (QIAGEN, Hilden, Germany) were conducted at 4, 6, 8, and 24 h post-infection. All extractions were treated with 2 units of DNase I (BioLabs, San Diego, CA, USA) and then purified using the Monarch^®^ RNA Cleanup Kit (New England BioLabs, Inc., Ipswich, MA, USA). One ug of RNA was used to produce cDNA using qScript cDNA SuperMix (Quanta bio, Beverly, MA, USA), which was used for qPCR. Primers and probes for the detection of the E111R gene were designed using the ASFV Georgia 2007/1 strain (GenBank Accession #NC_044959.2). Primer forward: 5′-GCGTATTACAATAGAGAATGAAGCAC-3′, reverse: 5′-TGTATCCACTCAAAAGCATTTTTATAAC-3′ and probe: 5′-FAM/TTAGCGCAGACCAGCACGTTGA/MGBNFQ-3′. Primers and probes for the detection of p72 gene: forward 5′-CTTCGGCGAGCGCTTTATCAC-3′, reverse: 5′-GGAAATTCATTCACCAAATCCTT-3′ and probe: 5′-FAM-CGATGCAAGCTTTAT-MGB NFQ-3′. Primers and probes for the detection of CP204L (p30) gene: forward 5′-GACGGAATCCTCAGCATCTTC-3′, reverse: 5′-CAGCTTGGAGTCTTTAGGTACC-3′ and probe: 5′-FAM-TGTTTGAGCAAGAGCCCTCATCGG-MGB NFQ-3′. Primers and probes for the detection of the β-Actin gene: forward 5′-GACCTGACCGACTACCTCATG-3′, reverse: 5′-TCTCCTTGATGTCCCGCAC-3′ and probe: 5′-FAM-CTACAGCTTCACCACCACGGC NFQ-3′. All qPCRs were conducted using the TaqMan Universal PCR Master Mix (Applied Biosystems, Waltham, MA, USA) using the following amplification conditions: one step at 55 °C for 2 min, followed by one denaturation step at 95 °C for 10 min, and then 40 cycles of denaturation at 95 °C for 15 s and annealing/extension at 65 °C for 1 min.

### 2.5. Evaluation of ASFV-G-ΔE111R Virulence in Pigs

The level of virulence of ASFV-G-ΔE111R was evaluated in 35–40 kg commercial-breed swine. Groups of pigs (n = 5) were intramuscularly (IM) inoculated with 10^2^ HAD_50_ of ASFV-G-∆E111R and compared with a similar group of pigs inoculated with 10^2^ HAD_50_ of ASFV-G. The presence of clinical signs associated with ASF (such as anorexia, depression, fever, purple skin discoloration, staggering gait, diarrhea, and cough) as well as body temperature values were daily recorded throughout the experiment. Blood samples were scheduled to be obtained at 0, 4, 7, 11, 14, 21, and 28 days post-inoculation (pi). Viremia titers were performed on primary swine macrophage cultures as described in Section 2.1. Experiments with pigs were performed under biosafety level 3 conditions in the animal facilities at Plum Island Animal Disease Center, following a strict protocol approved by the Institutional Animal Care and Use Committee (225.06-19-R_090716, approved on 6th September 2019).

## 3. Results and Discussion

### 3.1. Evolutionary Dynamics of E111R Gene of ASFV in Nature

**Figure 1:**
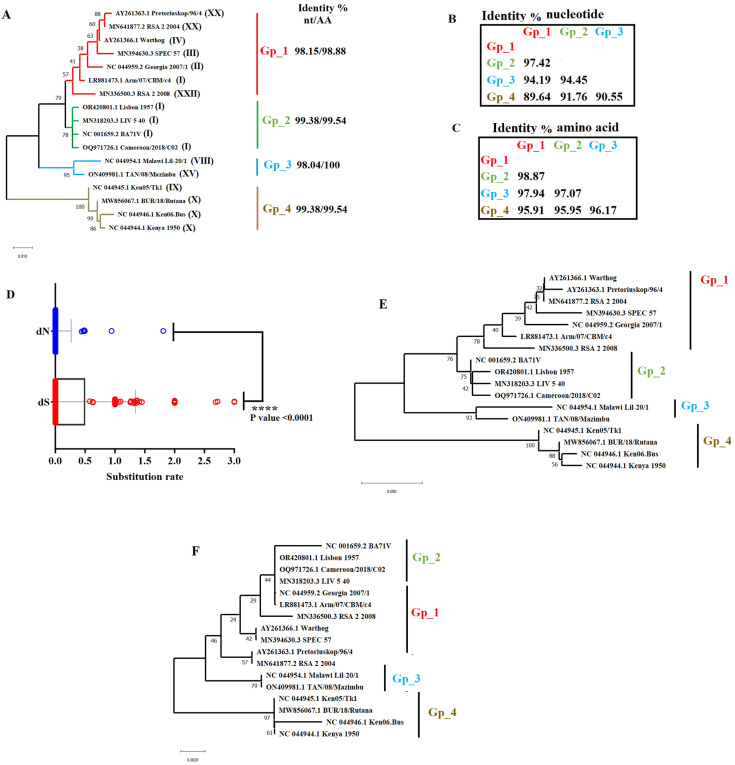
Phylogenetic dynamics of E111R gene in nature. (**A**) Phylogenetic analysis conducted by the neighbor-joining method using the full-length sequence of the E111R gene indicates the existence of four potential phylogenetic groups. Numbers in parentheses indicate the genotype of different strains based on p72 classification. Percentages of nucleotide (nt) and amino acid (AA) identities within groups are displayed. Pairwise distance analysis showing differences at the nucleotide (**B**) and amino acid levels (**C**) between phylogenetic groups are exhibited. (**D**) Comparison between synonymous (dS) and nonsynonymous (dN) substitution rates during the evolution of the E111R gene. Significant differences between dS and dN were determined by an unpaired t-test. The effect of dS and dN in shaping the phylogenetic relationship among ASFV isolates was assessed by the neighbor-joining method. Trees were reconstructed specifically by either synonymous (**E**) or nonsynonymous (**F**) mutations.

The ASFV E111R gene has been previously reported as being highly conserved among ASFV natural isolates [8]. In this report, we attempted to obtain more insights about the evolutionary dynamics behind the E111R gene of ASFV in nature. One of the first aspects considered in the evolution of E111R was to evaluate the phylogenetic relationship among natural ASFV isolates. For this purpose, we used the E111R gene of the Georgia_2007/1 isolate (GenBank access: NC_044959.2) to conduct a BLAST analysis to identify unique representative genotypes of this gene. As a result of this analysis, a total of 17 ASFV isolates associated with ten distinct genotypes (Based on p72 classification: I, II, III, IV, VIII, IX, X, XV, XX and XXII) were identified (Figure 1A). Then, using MEGA software (version 10.2.5) [15], we determined the Tamura 3-parameter + G (BIC score: 1860.723) to be the best substitution model for our phylogenetic analysis. The results of our phylogenetic analysis using the neighbor-joining method indicated that in nature, E111R has diverged in at least four phylogenetic groups (Gp_1 to Gp_4) (Figure 1A). These groups are composed of viral isolates that show high levels of identity within them at both the nucleotide and amino acid levels (Figure 1A). In this sense, the Georgia_2007/1 isolate, representative of genotype II and the current pandemic ASFV lineage [16], was liked to Gp_1, showing nucleotide similarities with isolates from diverse genotypes (I, III, IV, XX, and XXII). Furthermore, no differences at either the nucleotide or amino acid level were predicted among isolates associated with the pandemic lineage, indicating that E111R is not promoting the adaptation of this lineage in nature. Overall, consistent with the basal branch position of Gp_4 in the phylogenetic tree (Figure 1A), pairwise distance comparisons indicated that isolates Ken05/Tk1, BUR/18/Rutana, Ken06.Bus, and Kenya_1950 (Gp_4) represent the most dissimilar forms of E111R at both nucleotide and amino acid levels when compared with isolates from other groups (Figure 1B,C).

Next, we attempted to obtain more insights about the role of natural selection in the evolutionary dynamics of E111R in nature. Initially, as a general approach to infer the evolutionary pressures acting on E111R, we used the single-likelihood ancestor counting (SLAC) evolutionary algorithm [17]. Overall, dN/dS rates equal to 0.105 predicted by SLAC (calculated using the Global MG94xREV model), indicated that negative selection is the main evolutionary force acting on this gene, explaining the high conservation of the E111R protein previously reported in nature [8]. Furthermore, the low dN/dS value displayed by E111R was consistent with the significant (*p* < 0.0001) global differences predicted by SLAC between substitution rates at synonymous (dN) and nonsynonymous sites (dS) (Figure 1D), suggesting that synonymous substitutions accumulated ~10.68 times faster than nonsynonymous substitutions during the evolution of this gene in nature. Furthermore, when we compared the topology between phylogenetic trees constructed exclusively considering evolutionary distances at either synonymous sites (Figure 1E) or nonsynonymous sites (Figure 1F), we observed that the use of synonymous distances produced a topology similar to the tree displayed in Figure 1A, demonstrating the preponderance of synonymous mutations in shaping the phylogenetic relationships among ASFV isolates at the E111R genotype level.

**Figure 2:**
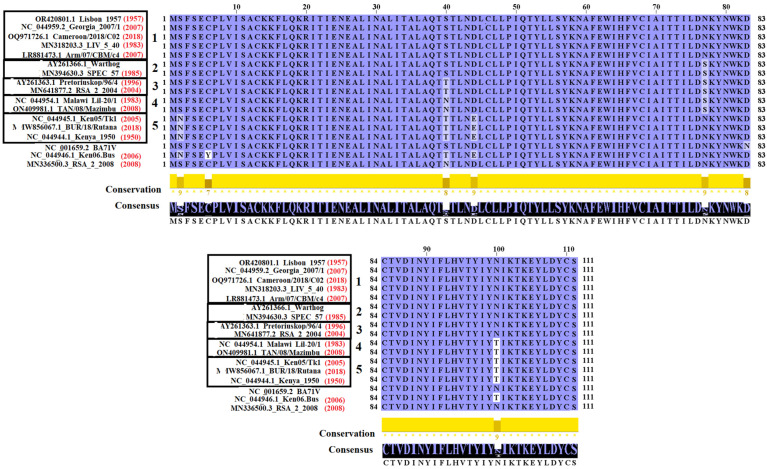
Grouping of ASFV isolates based on amino acid similarities in the E111R protein. Amino acid alignment showing similarities in the E111R protein among a group of 17 representative ASFV isolates. ASFV isolates within each box reflect isolates sharing 100% similarity. Five groups were identified. Red numbers in parenthesis indicate the year of collection of specific isolates (in some cases, no information was found). Conservation plot scores reflect the nature of the change at specific sites. Increased scores reflect substitutions between residues with similar biological properties. Analysis was conducted in the software Jalview version 2.11.1.7.

Based on the topology obtained by comparing nonsynonymous distances (Figure 1F), it was possible to regroup the isolates based on their 100% amino acid similarity (Figure 2). In this sense, a total of five groups of isolates were identified, while isolates BA71V, Ken06.Bus, and RSA2 2008 remained ungrouped, representing unique phenotypes. In the case of the isolate Georgia_2007/1 (representative of the pandemic lineage), it shares 100% amino acid similarity with multiple isolates associated with lineage I (Figure 2). Also, based on the collection year of some of these isolates, we can confirm the high stability of some of these phenotypes over time. It can be demonstrated in group 1 of the isolates that share 100% amino acid similarity with Lisbon 1957 (Figure 2); considering the collection year of this isolate (1957) and the one from the strain Cameron/2018/C02 (2018), it is possible to state that this phenotype has remained stable in nature for at least 61 years. Other examples of the tremendous stability of E111R in nature can be seen in groups 3, 4, and 5 (Figure 2), where these phenotypes have remained constant for at least 8, 25, and 68 years, respectively. The above highlights the relevance of negative selection in the evolutionary dynamics of E111R in nature.

**Figure 3:**
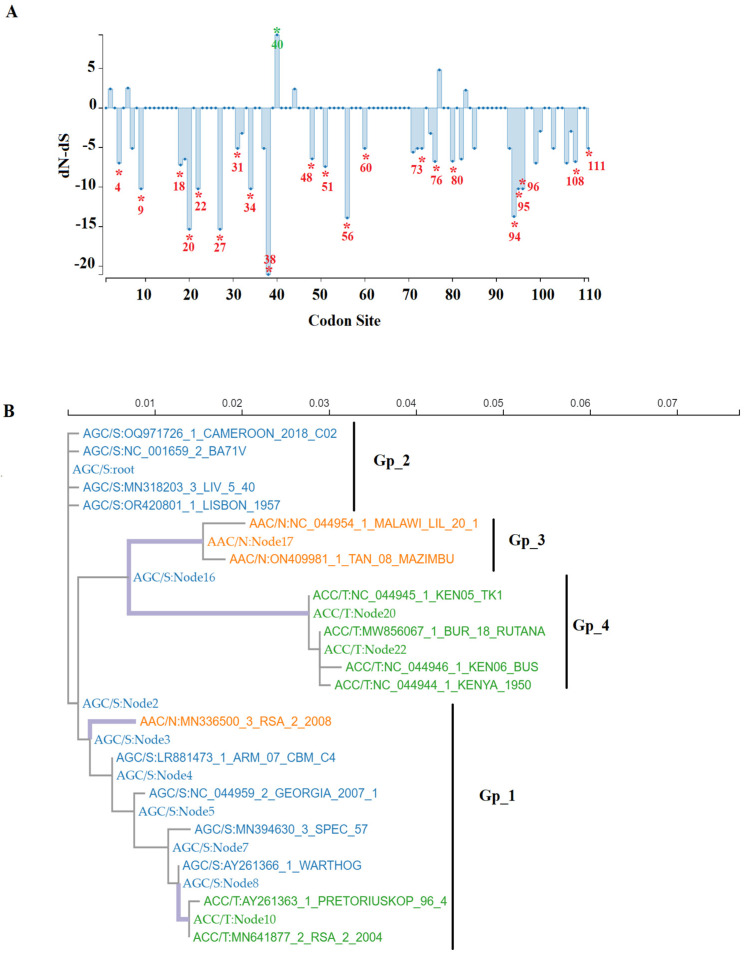
Evolutionary dynamics of the E111R gene in nature. (**A**) Graphic representation obtained by SLAC analysis, showing the ratio of dN-dS at specific codon sites in the E111R gene of ASFV. Identification of specific codon sites under positive selection (green asterisks) and negative selection (red asterisks) were obtained by FUBAR (posterior probability cutoff value = 0.9) and FEL (cutoff value of *p* = 0.1). Numbers close to the asterisk indicate the specific codon position. (**B**) Ancestral reconstruction analysis, showing the evolutionary dynamics of codon 40 of E111R gene. Predicted sequences at internal nodes (most probable common ancestor sequence associated with the divergence between and within genetic groups) and leaf nodes (represented by different isolates). Sequences in blue, green, and orange represent phenotypes (codon/amino acid) AGC/S, ACC/T, and AAC/N, respectively. Analysis was conducted using the mixed effects model of evolution algorithm (MEME) [18]. Results were saved in json format and visualized with the MEME analysis result visualization tool (https://observablehq.com/@spond/meme, accessed 25 April 2024).

Overall, no similarities have been predicted between the E111R protein and proteins previously reported from multiple data sources [8]. In our study, to predict potential functional sites preserved during the evolution of the E111R protein, we used the fixed effects likelihood (FEL) algorithm [17]. As a result, we identified a total of 21 codons evolving under negative selection (Figure 3A). The high conservation of the amino acids encoded by these codons strongly suggest their potential role in the functionality of the E111R protein. Furthermore, considering the minimal number of codons implied in nonsynonymous changes (codons: 2, 6, 40, 44, 77, 84, and 100) (Figure 3A), we then used the fast, unconstrained Bayesian approximation for inferring selection (FUBAR) evolutionary algorithm [18] to identify the potential role of these codons during the evolution of ASFV. FUBAR identified only codon 40 under positive selection (Figure 3A), implying that animo acid changes at this codon may promote the adaptation of ASFV in nature. To obtain more insights into the potential role of codon 40 in the adaptation of ASFV at the population level in nature, we conducted an ancestral reconstruction analysis using the maximum likelihood method. We detected diverse nonsynonymous changes at multiple internal nodes (predicted ancestral sequences) associated with the divergence between different genetic groups, suggesting that during the evolution of E111R in nature, multiple adaptive events were promoted by codon 40 (Figure 3B). In this sense, three distinct phenotypes were identified, including isolates carrying the codons/animo acids AGC/S (blue), ACC/T (green), and AAC/N (orange) (Figure 3B). The presence of the same codon within isolates in different genetic groups, suggest that parallel evolution may de associated in the adaptation promoted by codon 40 in nature [19]. Interestingly, FEL analysis conducted on internal nodes confirmed our last assumption, identifying codon 40 under positive selection (cutoff value of *p* = 0.1). This strongly suggests the role of codon 40 in the adaptation of ASFV at the population level. However, based on our limited knowledge regarding the functionality of the E111R protein, at this point, it is difficult to make any inferences about the possible implications of these results in the evolution of ASFV. Considering the virulent phenotype shown by ASFV-G-∆E111R in this and a previous study [8], it would be plausible to suggest the limited role of codon 40 in the virulence of ASFV. On the other hand, it is important to consider that the two studies conducted on ASFV lacking the E111R gene were carried out using parental viruses from the same lineage (Genotype II), and therefore future experimental studies are needed to confirm possible phenotypic differences in pigs among other E111R alleles present in ASFV. 

Overall, negative results from the genetic algorithm for recombination detection (GARD) [20] indicated that recombination does not play a role in the evolution of this gene.

### 3.2. Detection of E111R Transcription

**Figure 4:**
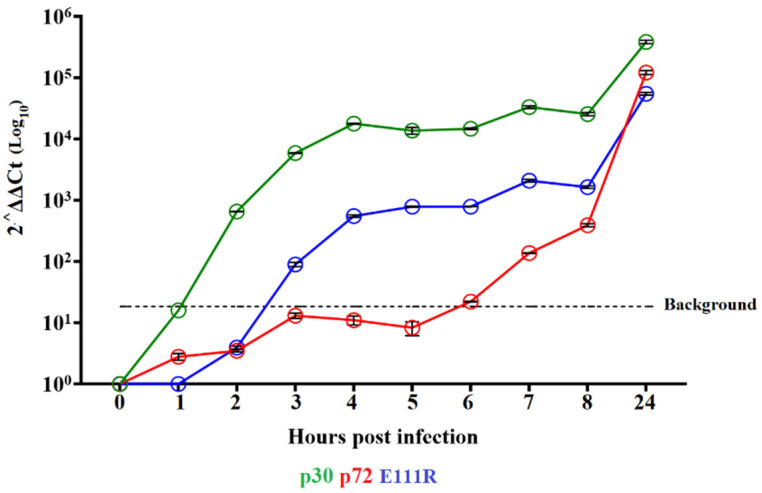
Expression profile of the E111R gene of ASFV during in vitro infection of porcine macrophages. Reverse transcription followed by qPCR was used to evaluate the expression profile of the E111R gene during in vitro infection at different time points, up to 24 h. As a reference for this analysis, we used qPCRs to specifically detect the expression of genes encoding ASFV proteins p30 (early expression) and p72 (late expression). Additionally, the β-Actin gene was used as a control to evaluate the quality and levels of RNA during the infection at different time points.

A timecourse experiment was performed to evaluate the kinetics of the E111R gene transcription during the virus replication cycle in primary swine macrophages infected with ASFV strain Georgia. Swine macrophage cultures were infected with ASFV-G at an MOI of 1, and cell lysate samples were taken at 4, 6, 8, and 24 hpi. The presence of E111R transcripts was detected (by two-step RT-PCR) as early as 4 hpi and persisted steadily until 24 hpi (Figure 4). The pattern of the transcription of the already well-characterized ASFV early proteins p30 (CP204L) and the late protein p72 (B646L) was used as a reference of early and late transcription profiles, respectively. The expression of E111R is similar to that of the p30 protein, indicating that the ASFV E111R gene encodes for a protein that is expressed early during the virus replication cycle. These results are in line with recently published studies in which the transcription kinetics of the E111R gene were analyzed in the SY18 isolate.

### 3.3. Development of the ASFV-G-ΔE111R Deletion Mutant

Because the high level of conservation in terms of nucleotide and amino acid sequence of the E111R gene among different ASFV isolates, it may be assumed that E111R may play an important role in the process of virus replication or virulence in natural hosts.

**Figure 5:**
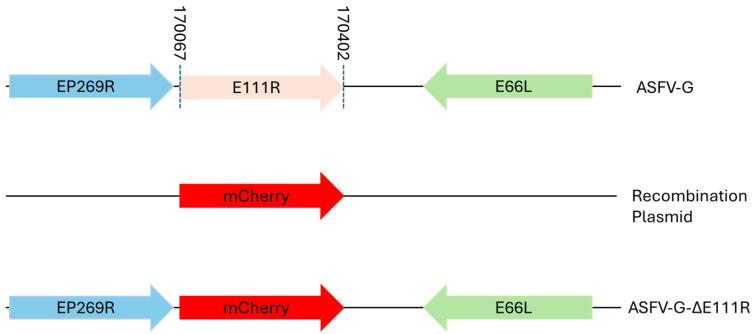
Schematic for the development of ASFV-G-∆E111R. The recombinant vector containing the mCherry reporter gene under the ASFV p72 promoter activity and the gene positions are shown. The nucleotide positions of the area that was deleted in the ASFV-G genome are indicated by the dashed lines. The resulting ASFV-G-∆E111R virus with the cassette inserted is shown on the bottom.

To evaluate the possible function of the E111R gene in virus replication in swine macrophages and in pigs, a recombinant virus with the E111R gene deleted was designed (ASFV-G-∆E111R) based on the highly virulent ASFV Georgia 2007 isolate (ASFV-G) as the parental virus. The E111R gene was deleted by replacing all 83 amino acid residues of the E111R ORF with the p72mCherry cassette by homologous recombination [7]. An area covering 249 bp (between nucleotide positions 170066 and 170401) was eliminated from the genome of ASFV-G, completely deleting the E111R gene, which was substituted with a 1226-bp cassette containing the p72mCherry construct (see Section 2) (Figure 5). The recombinant ASFV-G-∆E111R stock was purified by limiting dilution, using primary swine macrophage cell cultures as cell substrate.

The accuracy of the genetic modifications introduced into the ASFV-G-∆E111R genome was evaluated by obtaining the full genome sequence, NGS, using an Illumina NextSeq^®^ 550. The analysis of the results showed a deletion of 249 nucleotides and an insertion of 1226 nucleotides corresponding to the p72-mCherry cassette sequence. No unwanted additional genomic modifications were identified as developed during the development or the process of purification of ASFV-G-∆E111R. In addition, no evidence of the residual presence of the parental ASFV-G genome was detected as a potential contaminant in the ASFV-G-∆E111R stock.

### 3.4. Replication of ASFV-G-∆E111R in Primary Swine Macrophages

**Figure 6:**
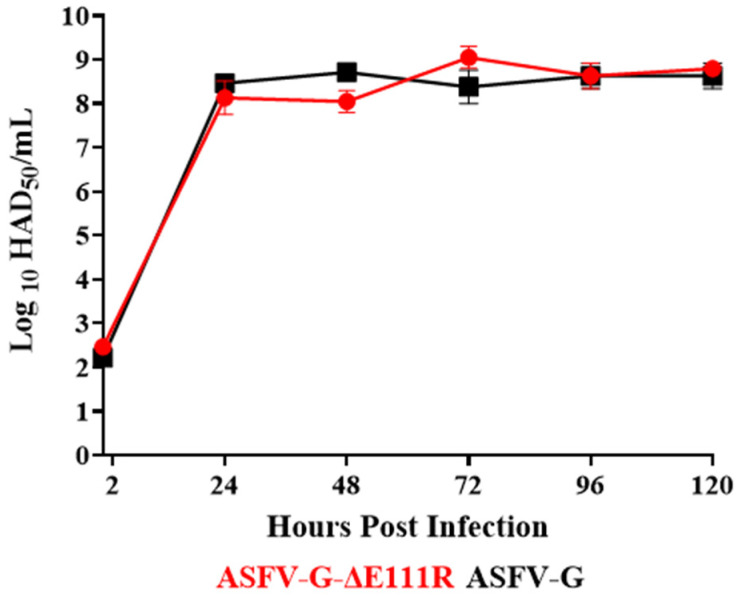
In vitro growth kinetics in primary swine macrophage cell cultures for ASFV-G-∆E111R and parental ASFV-G (MOI = 0.01). Samples were taken from two independent experiments at the indicated time points and titrated. Data represents means and standard deviations of two replicas. Sensitivity using this methodology for detecting virus is ≥log10 1.8 HAD_50_/mL.

To assess the probable function of the E111R gene in virus replication in swine macrophages, the capability of ASFV-G-∆E111R to replicate in swine macrophages was studied along with that of the parental ASFV-G using a multistep growth curve. Swine macrophages were infected (MOI = 0.01) with either ASFV-G-∆E111R or ASFV-G and virus titers determined at 2, 24, 48, 72, 96, and, 120 h post-infection (pi). ASFV-G-∆E111R and the parental ASFV-G showed almost overlapping kinetics with similar virus yields at all the sample points tested (Figure 6). Therefore, deletion of the AE111R gene from the genome of the ASFV-G did not affect virus replication in swine macrophages.

### 3.5. Assessment of ASFV-G-∆E111R Virulence in Swine

The potential effect of the E111R gene in the process of virulence of the ASFV-G isolate was evaluated on domestic pigs. The recombinant ASFV-G-∆E111R was experimentally inoculated in a group of pigs (n = 5) by the IM route at a dose of 10^2^ HAD_50_. As control, a group (n = 4) of animals was inoculated under the same conditions with the parental ASFV-G isolate. The presence of clinical signs in both animal groups were planned to be observed for 28 days.

**Figure 7:**
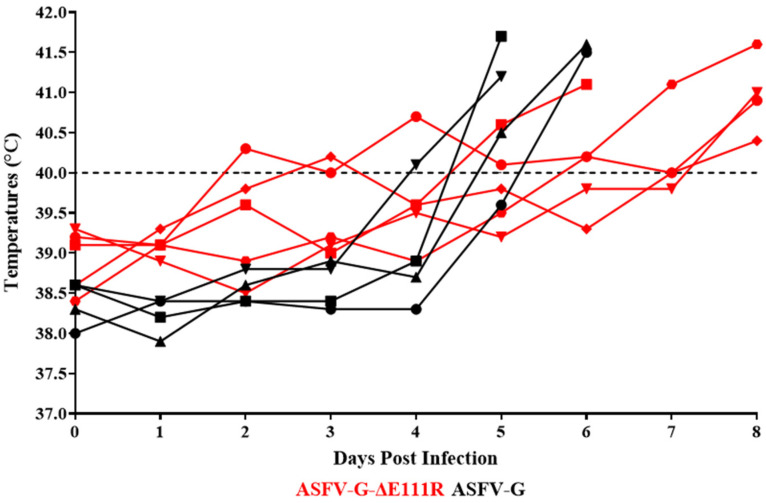
Evolution of body temperature in animals (5 animals/group) IM infected with 10^2^ HAD_50_ of either ASFV-G-∆E111R or parental ASFV-G.

**Figure 8:**
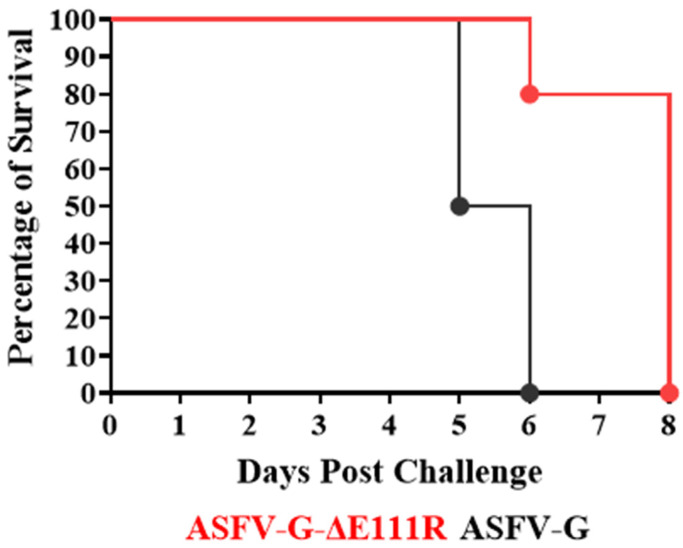
Evolution of mortality in animals IM infected with 10^2^ HAD_50_ of either ASFV-G-∆E111R or parental ASFV-G.

As expected, the pigs experimentally inoculated with the virulent parental ASFV-G presented an increase in body temperature (>40 °C) by day 4–5 pi, rapidly evolving to full clinical disease (depression, anorexia, staggering gait, diarrhea, and purple skin discoloration) (Figure 7 and Figure 8), with all animals being euthanized by day 5–6 pi.

The animals inoculated with ASFV-G-∆E111R developed a lethal form of the disease similar to those inoculated with ASFV-G but with slightly protracted kinetics. Animals started showing an increase in body temperature over 40 °C on days 4 to 6 post-infection. Clinical disease quickly worsened, with all animals needing to be euthanized between days 6 and 8 post-infection. These results indicate that deletion of the E111R gene from the genome of ASFV-G does not produce a significant decrease in virus virulence in experimentally infected domestic swine.

**Figure 9:**
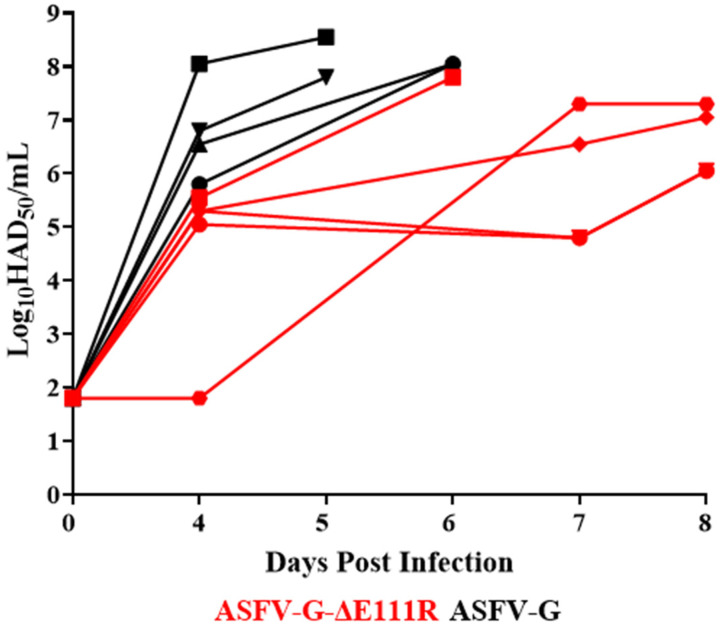
Viremia titers detected in pigs IM inoculated with 10^2^ HAD_50_ of either ASFV-G-∆E111R or ASFV-G. Each symbol represents individual viremia titers in each animal in the groups. Sensitivity of virus detection: ≥log10^1.8^ TCID_50_/mL.

The replication ability of ASFV-G-∆E111R in the inoculated animals was assessed by quantifying viremia after experimental inoculation and comparing that with viremias observed in the animals inoculated with the parental virulent ASFV-G (Figure 9). Animals inoculated with ASFV-G presented high viremia titers (ranging from 10^5.55^–10^8^ HAD_50_/mL) at day 4 pi, lasting with high values (ranging from 10^7.55^–10^8.2^ HAD_50_/mL) until day 5 or 6 pi when all animals were euthanized. Viremia titers in pigs receiving ASFV-G-∆E111R showed similar but slightly protracted kinetics than those animals inoculated with the parental virus. All animals but one showed titer values between 10^4.05^–10^5.55^ HAD_50_/mL by day 4 pi, with all animals’ viremias slightly increasing (ranging from 10^4.8^–10^7.8^ HAD_50_/mL) by day 7 or 8 pi, when they needed to be euthanized due to the severity of clinical signs. It is important to note that at necropsy, blood samples were taken from all five animals inoculated with ASFV-G-∆E111R, and the virus was present in each of samples sequenced by NGS. Analysis of the full-length genome of each of these viruses demonstrated that ASFV-G-∆E111R was (and not a potential cross contamination with ASFV-G) responsible for producing the lethal form of the disease observed in these animals. In addition, no unexpected genomic modifications (mutations or genomic rearrangements) were observed in the genome of the viruses isolated from the ASFV-G-∆E111R-inoculated animals. Therefore, ASFV-G-∆E111R developed a systemic infection in experimentally inoculated animals that resembles that induced by the parental ASFV-G, but appearing slightly decreased and protracted.

Results presented here clearly indicate that the deletion of E111R gene does not affect basic ASFV functions regarding virus replication in macrophages (both in cell cultures and pigs experimentally infected) and the virulence of ASFV strain Georgia in domestic pigs.

Previously, Zhou et al. [8] deleted the E111R gene in the Chinese ASFV virulent field strain SY18 (SY∆E111R), resulting in a later disease onset of ASFV and 40% of the animals surviving when experimentally inoculated at a dose of 10^2^ TCID_50_. In our study, deletion of E111R did not produce any significant difference in clinical disease between parental ASFV-G and ASFV-G-∆E111R. Although the deletion designs of the E111R gene were technically the same, the selection reporters to make both recombinants were different. In Zhou et al. [8] study, a SV40polyA was added to the EGFP cassette, and in our study, we only included an ASFV terminator sequence. In addition, the SY18 virus was grown in bone marrow-derived cells and the ASFV-G was always passed in blood-derived primary swine macrophages for its development and purification. Although neither of these variables have been reported to be relevant in the induction of phenotypic changes in ASFV recombinant viruses, these are the only differences in the production of the recombinant viruses lacking E111R in these two reports.

Interestingly, in the SY18 study, parental SY18 swine had a mean time till death of 9 days after the IM inoculation of 10^2^ TCID_50_, while in our study, animals inoculated with the same dose of parental ASFV-G had a mean time of death of 5.5 days after inoculation, suggesting that the ASFV-G strain could be more virulent than SY18 and that could be the reason for the differences in the two studies, particularly with increasing doses in SY∆E111R showing the same phenotype as in ASFV-G-∆E111R. Importantly, the only observed genetic differences between SY18 and ASFV-G stretches of nucleotide repeats, an insertion that occurs at position 173398 in the SY18 sequence that is not present in the ASFV-G sequence. It should be mentioned here that the ends of the SY18 genome have not been fully resolved; however these differences could be purely due to different sequencing protocols and analysis of these genomes. Moreover, this difference does not seem to affect protein coding regions. Therefore, is difficult to identify the reasons behind the differences in the results obtained in the experimental infection using low doses between these two reports. This is another example in the field of ASFV that standardized methods for evaluating ASFV pathogenesis are needed, to ensure reproducibility between groups.

In addition, it has been frequently reported the production of heterogeneous phenotypes by deleting specific gene/s (even some of them highly conserved across isolates) from the genome of different virus isolates. Differences in the effect on virulence in domestic pigs of recombinant viruses having deletions in some highly conserved ASFV genes as 9GL, UK, DP148R, EP402R, TK have been reported by several research groups [21,22,23,24,25] although the genetic bases explaining those results are still unidentified. Therefore, differences in the results reported here and by Zhou et al. [8] regarding the deletion of the E111R gene may be similar reasons. In summary, we present here a detailed analysis of the evolutionary dynamics of E111R gene of ASFV in nature, determined that E111R is a non-essential gene, since its deletion from the ASFV-G genome does not significantly alter virus replication in swine macrophage cultures, and at least in the ASFV Georgia isolate E111R gene is not involved in the process of virus virulence in the domestic pigs.

## Data Availability

All data are included in the manuscript.

## References

[1] Gonzales W., Moreno C., Duran U., Henao N., Bencosme M., Lora P., Reyes R., Nunez R., De Gracia A., Perez A.M. (2021). African swine fever in the Dominican Republic. Transbound. Emerg. Dis..

[2] Tulman E.R., Delhon G.A., Ku B.K., Rock D.L., Etten V. (2009). African Swine Fever Virus. Lesser Known Large dsDNA Viruses.

[3] Gladue D.P., Borca M.V. (2022). Recombinant ASF live attenuated virus strains as experimental vaccine candidates. Viruses.

[4] Borca M.V., Ramirez-Medina E., Silva E., Vuono E., Rai A., Pruitt S., Espinoza N., Velazquez-Salinas L., Gay C.G., Gladue D.P. (2021). ASFV-G-I177L as an Effective Oral Nasal Vaccine against the Eurasia Strain of Africa Swine Fever. Viruses.

[5] Borca M.V., Ramirez-Medina E., Silva E., Vuono E., Rai A., Pruitt S., Holinka L.G., Velazquez-Salinas L., Zhu J., Gladue D.P. (2020). Development of a Highly Effective African Swine Fever Virus Vaccine by Deletion of the I177L Gene Results in Sterile Immunity against the Current Epidemic Eurasia Strain. J. Virol..

[6] Gladue D.P., Ramirez-Medina E., Vuono E., Silva E., Rai A., Pruitt S., Espinoza N., Velazquez-Salinas L., Borca M.V. (2021). Deletion of the A137R Gene from the Pandemic Strain of African Swine Fever Virus Attenuates the Strain and Offers Protection against the Virulent Pandemic Virus. J. Virol..

[7] O’Donnell V., Holinka L.G., Krug P.W., Gladue D.P., Carlson J., Sanford B., Alfano M., Kramer E., Lu Z., Arzt J. (2015). African swine fever virus Georgia 2007 with a deletion of virulence-associated gene 9GL (B119L), when administered at low doses, leads to virus attenuation in swine and induces an effective protection against homologous challenge. J. Virol..

[8] Zhou X., Fan J., Zhang Y., Yang J., Zhu R., Yue H., Qi Y., Li Q., Wang Y., Chen T. (2023). Evaluation of African Swine Fever Virus E111R Gene on Viral Replication and Porcine Virulence. Viruses.

[9] Borca M.V., Berggren K.A., Ramirez-Medina E., Vuono E.A., Gladue D.P. (2018). CRISPR/Cas Gene Editing of a Large DNA Virus: African Swine Fever Virus. Bio-Protocol.

[10] Krug P.W., Holinka L.G., O’Donnell V., Reese B., Sanford B., Fernandez-Sainz I., Gladue D.P., Arzt J., Rodriguez L., Risatti G.R. (2015). The progressive adaptation of a georgian isolate of African swine fever virus to vero cells leads to a gradual attenuation of virulence in swine corresponding to major modifications of the viral genome. J. Virol..

[11] Vuono E.A., Ramirez-Medina E., Pruitt S., Rai A., Espinoza N., Velazquez-Salinas L., Gladue D.P., Borca M.V. (2021). Evaluation of the Function of the ASFV KP177R Gene, Encoding for Structural Protein p22, in the Process of Virus Replication and in Swine Virulence. Viruses.

[12] Reed L.J., Muench H. (1938). A simple method of estimating fifty percent endpoints. Am. J. Hyg..

[13] O’Donnell V., Risatti G.R., Holinka L.G., Krug P.W., Carlson J., Velazquez-Salinas L., Azzinaro P.A., Gladue D.P., Borca M.V. (2017). Simultaneous deletion of the 9GL and UK genes from the African swine fever virus Georgia 2007 isolate offers increased safety and protection against homologous challenge. J. Virol..

[14] Borca M.V., O’Donnell V., Holinka L.G., Sanford B., Azzinaro P.A., Risatti G.R., Gladue D.P. (2017). Development of a fluorescent ASFV strain that retains the ability to cause disease in swine. Sci. Rep..

[15] Kumar S., Stecher G., Li M., Knyaz C., Tamura K. (2018). MEGA X: Molecular Evolutionary Genetics Analysis across Computing Platforms. Mol. Biol. Evol..

[16] Gallardo C., Soler A., Nurmoja I., Cano-Gomez C., Cvetkova S., Frant M., Wozniakowski G., Simon A., Perez C., Nieto R. (2021). Dynamics of African swine fever virus (ASFV) infection in domestic pigs infected with virulent, moderate virulent and attenuated genotype II ASFV European isolates. Transbound. Emerg. Dis..

[17] Kosakovsky Pond S.L., Frost S.D. (2005). Not so different after all: A comparison of methods for detecting amino acid sites under selection. Mol. Biol. Evol..

[18] Murrell B., Wertheim J.O., Moola S., Weighill T., Scheffler K., Kosakovsky Pond S.L. (2012). Detecting individual sites subject to episodic diversifying selection. PLoS Genet..

[19] Escalera-Zamudio M., Golden M., Gutierrez B., Theze J., Keown J.R., Carrique L., Bowden T.A., Pybus O.G. (2020). Parallel evolution in the emergence of highly pathogenic avian influenza A viruses. Nat. Commun..

[20] Kosakovsky Pond S.L., Posada D., Gravenor M.B., Woelk C.H., Frost S.D. (2006). Automated phylogenetic detection of recombination using a genetic algorithm. Mol. Biol. Evol..

[21] Chen W., Zhao D., He X., Liu R., Wang Z., Zhang X., Li F., Shan D., Chen H., Zhang J. (2020). A seven-gene-deleted African swine fever virus is safe and effective as a live attenuated vaccine in pigs. Sci. China Life Sci..

[22] Rathakrishnan A., Reis A.L., Goatley L.C., Moffat K., Dixon L.K. (2021). Deletion of the K145R and DP148R Genes from the Virulent ASFV Georgia 2007/1 Isolate Delays the Onset, but Does Not Reduce Severity, of Clinical Signs in Infected Pigs. Viruses.

[23] Monteagudo P.L., Lacasta A., Lopez E., Bosch L., Collado J., Pina-Pedrero S., Correa-Fiz F., Accensi F., Navas M.J., Vidal E. (2017). BA71∆CD2: A New Recombinant Live Attenuated African Swine Fever Virus with Cross-Protective Capabilities. J. Virol..

[24] Borca M.V., Carrillo C., Zsak L., Laegreid W.W., Kutish G.F., Neilan J.G., Burrage T.G., Rock D.L. (1998). Deletion of a CD2-like gene, 8-DR, from African swine fever virus affects viral infection in domestic swine. J. Virol..

[25] Ramirez-Medina E., Vuono E., O’Donnell V., Holinka L.G., Silva E., Rai A., Pruitt S., Carrillo C., Gladue D.P., Borca M.V. (2019). Differential Effect of the Deletion of African Swine Fever Virus Virulence-Associated Genes in the Induction of Attenuation of the Highly Virulent Georgia Strain. Viruses.

